# Optimization and characterisation of water based keratin nanoparticles with *Nigella sativa* seed extract

**DOI:** 10.6026/973206300200957

**Published:** 2024-09-30

**Authors:** Lawrance Antonysamy, Leema Rose Mary Devasahayam, Saradhadevi Muthukrishnan, Senthamizhselvan Anbazhagan, Marie Arockianathan Pushpam

**Affiliations:** 1 PG and Research Department of Biochemistry, St.Joseph's College of Arts and Science, (Autonomous), Cuddalore-607001, Tamil Nadu, India; 2 Department of Biochemistry, Bharathiar University, Coimbatore, Tamil Nadu, India

**Keywords:** *Nigella sativa*, chicken feather, keratin, drug delivery, nanoparticles

## Abstract

Drug delivery technologies have been proven to improve treatment outcomes in many ways by enriching curative efficacy, reducing toxicity, increasing patient compliance and enabling entirely new type of medical treatments. In this study, keratin nanoparticles (KNPs) were prepared from chicken feathers and loaded with *Nigella sativa* seed extract (NSSE-KNPs) by adsorption method using doubled distilled water without using cross linkers, organic solvents and surfactants. The prepared KNPs and NSSE-KNPs were characterized by Dynamic light scattering (DLS), X-ray diffraction study (XRD), Fourier transform infrared spectroscopy (FT-IR) and scanning electron microscopy (SEM). The Encapsulation efficiency (EE) and loading capacity (LC) of NSSE-KNPs was found to be 82% and 70 % respectively. The drug release profile showed that KNPs exhibited slight difference in both acidic (pH 4.4) and basic (pH 7.4) environment. The prepared water-based KNPs and NSSE-KNPs also exhibited narrow PDI value and good negative *zeta potential*. The morphology of the keratin nanoparticles and *Nigella sativa* loaded keratin nanoparticles showed solid spheres, spherical and smooth shape distribution. The FTIR spectra revealed the possible hydrogen bonding formation after addition *Nigella sativa* seed extract to the keratin nanoparticles. In XRD analysis, both KNPs and NSSE-KNPs retained its chemical structure and crystallinity. The antibacterial effect was also observed for NSSE-KNPs against *Staphylococcus aureus* and *Escherichia coli*. Thus, the keratin nanoparticles loaded NSSE extract emerged as a potential candidate for future cancer treatment.

## Background:

Over the past several decades, the field of drug delivery has increased dramatically in different ways. Several new synthetic drug delivery systems have been designed and developed by bioengineers with sustained release and targeted delivery to improve their identifications and treatment of diseases [1]. Synthetic polymers have greater advantages over natural polymers due to their wide range of properties, but the toxicity, accumulation in organs, and the side effects make it easier to reach of alternative. The alternative biodegradable natural biopolymers have attracted researchers in the area of controlled drug delivery systems because of its safer use than synthetic polymers [2]. Biopolymers are obtained from plants and animals like starch, lignin, cellulose, alginate and agarose in plants and in animal's collagen, gelatin, silk, fibrin, chitin, chitosan and keratin. These biopolymers were incorporated into various polymers which had enormous applications in the fields of polymer chemistry, molecular and cellular biology [3]. These biopolymers possess some unique features such as biodegradability, biocompatibility and remodeling properties, that they have been tested for tissue engineering applications as skin scaffolds, peri-implant dental tissue regeneration, bone graft, hydrogel-based cartilage, dermal bio fillers and for drug delivery systems [4]. Moreover, these polymers do not accumulate in organs as metal nanoparticles (or) synthetic polymers [5]. Among the natural polymers, protein based polymers exhibited great potential for biological applications. Keratin was one of the biopolymer derived from various natural sources like corneum of the epidermis and epidermal appendages such as hair, wool, feathers, fingernails, animal claws and horn [6]. Among the sources, chicken feathers waste created lot of havoc to environment pollution. So, from this waste, keratin was extracted as it possesses a unique amino acid sequence that gives support to the material. Keratin was mostly explored as a drug carrier, as it improve therapeutic effects and reduced side effects by encapsulating with other drugs. Recently, keratin- based biomaterials have drawn much attention due to their excellent properties like structural versatility, biodegradability, non-toxicity, high porosity, mechanical durability, biocompatibility, biosorbent, self-assembly, intrinsic cellular recognition and high availability [7].

Keratin was mostly explored as a drug carrier, as it improves therapeutic effects and reduced side effects by encapsulating drugs. Recently, keratin- based biomaterials have drawn much attention due to their excellent properties like structural versatility, biodegradability, non-toxicity, high porosity, mechanical durability, biocompatibility, biosorbent, self-assembly, intrinsic cellular recognition and high availability [7]. Keratin has molecular structures similar to collagen, a prominent protein in native extracellular matrices (ECMs). The presence of tripeptides "Arg- Gly - Asp (RGD) and Leu -Asp- Val (LDV), that specifically bind cell surface receptor vitronectin, integrin and promote cell adhesion and support cell recognition sites on keratin-based materials. The most extensively explored applications of keratin as a drug carrier, involves the encapsulation of drugs into keratin-based nanoparticles to improve therapeutic effects and reduced side effects of drugs [8]. Due to its fascinating property, keratin nanoparticles (KNPs) found to have many biological and environmental applications, and making them suitable for wound healing, tissue engineering, and cancer treatment, removal of hazardous substances from wastewater and bimolecular detection and diagnosis.

Several literature have been reported the use keratin nanoparticles for drug delivery [9] or in combination with metals such as silver, gold [10], anti-tumoral drugs such as, paclitaxel [11], doxorubicin[12], docetaxel [13], and 9(R)-9-hydroxy stearic acid[14], and with synthetic polymers such as polyethylene glycol (PEG), poly acrylonitrile (PAN), polyvinyl chloride (PVS), poly lactic acid (PLA),polyamide-6 (PA6), and poly caprolactone (PCL) and so on [15,16,17]. In addition, keratin nanoparticles incorporated with natural polymers such as cellulose, chitosan, alginate, starch, collagen and gelatin, etc. have been reported in drug delivery systems [18]. Nowadays medicinal plants were utilized for treating several diseases such as infections, cardiovascular diseases asthma and gastrointestinal problems. Among various medicinal plants, *Nigella sativa* (*N.Sativa*), commonly known as black cumin, has been used as a spice, food preservative, and also as a protective, curative remedy for several disorders [19]. The powder, paste, and essential oils, prepared from the seed of *Nigella sativa* has been used in traditional medicine for many diseases/conditions such as headache, asthma, back pain, inflammation, amenorrhea, bronchitis, anorexia, and hypertension etc. [20]. *Nigella sativa* seeds have a lot of benefits due to the presence of many bioactive constituents such as thymoquinone (TQ), P- cymene, carvacrol, thymohydroquinone (THC), dihydro thymoquinone (DHTO), saponin, alpha -thujene, thymol, beta-pinene, alpha - pinene and gamma-terpinene [21], which were mostly responsible for its pharmacological and therapeutic effects. These bioactive compounds have been shown to possess a wide range of activities, including anticancer, diuretic, analgesic, anti-diabetic, antihypertensive, immunomodulatory, anti-inflammatory, anti-helminthic, hepato protective, renal protective, neuroprotective, antibacterial, bronchodilator, and antioxidant properties [22]. Recent report showed the incorporated keratin, extracted from chicken feathers, into ginger starch to produce bio-composite film showed an extraordinary biocompatibility in drug delivery system [23]. PEGlyated -TQ nanoparticles were used to retard the migration of breast cancer cells and also thymoquinone (TQ) combined with doxorubicin showed anticancer properties against MCF cell lines [24]. In the present study, a novel water-based Keratin nanoparticles were prepared (KNPs) by ultrasonic dispersion method and loaded with *Nigella sativa* seed extract (NSEE). The prepared water based NSSE-KNPs and KNPs were physiochemically characterized. The release studies were evaluated at different pH to study their release kinetics, and it also tested for its antibacterial properties.

## Materials and Methods:

## Materials:

Seeds of *Nigella sativa* were obtained from Nilgiris store in Puducherry, India. Broiler Chicken feathers were collected from poultry house in Cuddalore. Sodium sulfide, Sodium hydroxide, Hydrochloric acid, petroleum ether and formaldehyde were purchased from Merck. Pvt. Ltd. All other chemicals used in this study were of analytical grade.

## Methods:

## Preparation of keratin nano particles (KNP):

Keratin extraction was done from the boiler chicken feathers by using sodium sulfide at pH 4.7 [25]. The extracted keratin powder was washed several times to become neutral. The powder was then lyophilized and dissolved in saline (pH 7.4) at different propositions (1, 2 and 4 mg/mL). To further break the keratin micro particles into nanoparticles, micro particles were sonicated using ultrasonic cell disruption system with 40-50% amplitude. Finally, the obtained KNPs was lyophilized and stored.

## Extraction of *Nigella sativa* seed extract:

The procured *Nigella sativa* seeds were washed with tap water thrice to clean the dust particles and air dried for one week under partial darkness. The seeds were then grounded into fine powder. 20 gm of powdered seeds were used for extraction using dichloromethane as solvent in soxhlet apparatus for 6 hours at 55-65 °C. The obtained extract was transferred to another flask and the solvent was evaporated using rotary evaporator at 45°C for 15min, and stored at 4°C for further analysis.

## Preparation of water-based *Nigella sativa* seed extract loaded with Keratin nano particles (NSSE-KNPs):

The prepared keratin nanoparticles were first dissolved in water (pH 7.4) at different propositions (1mg/mL, 2mg/mL and 4mg/mL). The solubility of keratin in water was significantly enhanced by the addition of base to make it mild alkaline. To this, a series of *Nigella sativa* seed extract loaded with keratin nanoparticles (NSSE-KNPs) were prepared by using double distilled water at different concentrations. The above solution was constantly stirred at 700 rpm for 60 minutes at 40°C. The obtained *Nigella sativa* seed extract loaded keratin nanoparticles (NSSE-KNPs) were lyophilized and kept for further studies. The yield%, drug loading capacity (LC) and drug encapsulation efficiency (EE) was calculated [26] according to the following equations

Total yield (%) = Total weight of nanoparticles and drug (mg) / Actual weight of the product (mg) x 100

Loading efficiency (%) = Weight of drug in nanoparticles (mg) / Weight of initially added nanoparticles (mg) x 100

Loading capacity (%) = Weight of drug in nanoparticles (mg) / Weight of drug (extract) + loaded nanoparticles (mg) x 100

## Characterization of keratin nanoparticles (NPs) and *Nigella sativa* extract (NSSE-KNPs) loaded Keratin nanoparticles:

## *Particle size* and *zeta potential* (ZP):

The size measurement of keratin nano particles and *Nigella sativa* seed extract loaded keratin nanoparticles was done by using Malvern Zetasizer Nano 2590 System, in which freeze -dried nanoparticles were dispersed in different pH of Phosphate buffer solution. The polydispersity index (PDI) of the *Particle size* and *zeta potential* was measured.

## SEM-Scanning Electron Microscopy (SEM):

The surface morphology of the lyophilized dried keratin nanoparticles (KNPs) and *Nigella sativa* seed extract loaded keratin nanoparticles (NSSE-KNPs) were studied using Scanning Electron microscopy (TSCAN VEGA 3) at an accelerating voltage of 10-20 kV. Prior to examination, the samples were placed on an adhesive stub and coated with gold under vacuum for 45s using a Joel-JFC 1600 auto file coated system.

## FTIR-Fourier transforms infrared spectroscopy:

The Perkin Elmer Spectrum 100 Germany FT-IR spectrometer was used to evaluate the chemical nature of freeze dried keratin nanoparticles and *Nigella sativa* seed extract loaded keratin nanoparticles with frequency range of 500-4000 cm^-1^. The samples were specifically analyzed to identify organic groups and compounds with various side chains, cross links, and functional groups [27].

## X-ray diffraction analysis:

The X-ray diffraction studies was also conducted to determine the chemical changes of keratin nanoparticles and Nigella saliva seed extract loaded keratin nanoparticles using an X-ray diffractometer (Bruker USA D8Focus, Davinci model). Diffraction intensities were documented in 2-theta ranging from 10°-80° to determine the chemical changes (level of crystallinity) in the keratin samples.

## *In-vitro* drug release studies:

Dissolution studies were conducted using a Mx 7L heating bath. 100 mg of *Nigella sativa* seed extract (NSSE-KNPs) loaded keratin nanoparticles dissolved in 5 ml buffer solution was placed into receptor compartment containing 3.5 ml dissolution medium, which was shaken gently at 37±0.5 °C. The receptor compartment was closed to prevent the evaporation losses from the dissolution medium. Periodically 3.5 ml of release medium was withdrawn, and 3.5 ml of fresh PBS was added to the system. The *Nigella sativa* seed extract concentration in the supplied medium was determined by UV spectrometer with absorption wavelength at 276nm [28].

## Antibacterial susceptibility test:

Mueller-Hinton agar was used in the agar well diffusion method to test for antibacterial sensitivity. The bacterial strains used in this study were *Staphylococcus aureus*, *Escherichia coli*. The culture of *Staphylococcus aureus*, *Escherichia coli* kept overnight was used to inoculate Muller-Hinton agar plate using sterile cotton swab. Using a sterile cork borer, wells were made on seeded plates measuring 6 mm in diameter. Using a micropipette, keratin nanoparticles and *Nigella sativa* seed extract loaded keratin nanoparticles added each to two well at varying concentrations (5 and 10µL) in same plate. The plates were then incubated at °C for 24 hours in an upright position and the zone of inhibition was observed.

## Results & Discussions:

## *Particle size*, size distribution and *zeta potential*:

The *Particle size* analysis plays a vital role in drug delivery to calculate different properties of nanoparticles and to identify its interaction between other substances under certain conditions. The suitability of nanoparticles formulations for a particular route of drug administration depends on their average diameter, PDI and size stability. The smaller size of the particles exhibit larger surface area, thereby have more surface area to interact with other molecules (or) cells, flow ability and also have more retention time.

**Figure 1:**
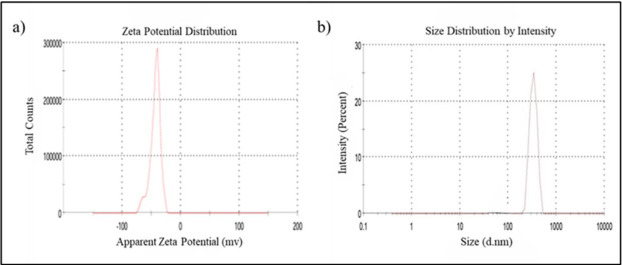
Z-potential (a) and particle size distribution (b) of Keratin nanoparticles (KNPs)

**Figure 2:**
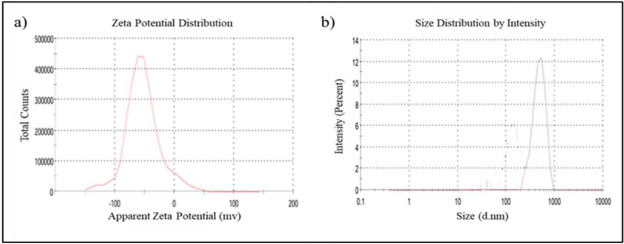
Z-potential (a) and *particle size* distribution (b) of *Nigella sativa* seed extract loaded Keratin nano particles (NSSE-KNPs).

Among the three different formulations, the keratin nanoparticles (KNPs) with 2 mg/mL concentrations showed smaller size (338.2nm) and lower PDI (0.410) value (Figure 1). The PDI value less than 0.5 showed greater particle stability for nano delivery and showed uniform homogenous *Particle size* distribution in colloidal suspensions [29]. This concentration clearly indicated the more dispersive nature, greater particle stability and homogenous in nature. This may be due to the sonication method than the manual method used. The PDI value was used to analyse the nano delivery system performance and its uniformity. The narrow size distribution of nanoparticles will have low PDI value. This characteristic property gave a consistent performance of the drug delivery system by controlling drug release kinetics, uptake by cells and its targeted location. So, the PDI value was used to get desired beneficial effects and also lessen its side effects. KNPs of 2mg/mL concentration showed *zeta potential* value-43.9 and the PDI value 0.410 (Figure 1). This indicated that KNPs were stable due to its high negative charge and narrow PDI value. The size distributions of KNPs were found to be narrow size distribution of 338.2nm. So, the keratin nanoparticles (2mg/ml) as taken for loading NSSE and for further characterization. The size, PDI and *zeta potential* of NSSE (1.8mg) loaded keratin nanoparticles (2mg/mL) were depicted in Table 1. In our results, we observed that the size was increased slightly in NSSE-KNPs (437.5nm) than the unloaded keratin nanoparticles (338.2 nm) by DLS method (Table 1).These correlates with previous report which showed that the drug doxorubicin loaded keratin nanoparticles size were increased slightly when compared with unloaded keratin nanoparticles [30]. In our results, the increase in size was mainly due to the strong electrostatic interaction between NSSE and the deprotonation of keratin polymer, indicating efficient encapsulation of Nigella saliva seed extract with keratin nanoparticles. *zeta potential* (ZP) was an important parameter to identify the stability and the nature of the nanoparticles in drug delivery systems. Higher negative or positive values of ZP prevent aggregation of NPs by their repulsive force. The negative *zeta potential* of KNP was mainly due to the functional group S-carboxymethyl on its surface, pH of the medium and by the ions absorbed. The ZP of the prepared KNPs was -43.9, and for NSSE-KNPs was -53.0. This negative charge on the surface of the KNPs will prevent aggregation of the KNPs thereby maintain the strong repulsive force among the particles [31] and thereby maintaining dispersive nature in its suspension. The increase in size of NSSE-KNPs (437.5nm) was due to strong interaction between NSSE and carboxyl groups in keratin nano particles (Figure 2).

## Encapsulation efficiency (EE), loading capacity (LC) and yield (%):

The three different concentration of keratin nanoparticles (KNPs) (1 mg, 2mg and 4mg) were loaded with different concentration of *Nigella sativa* seed extract with 900mg, 1.8g and 3.6g respectively. The Encapsulation efficiency of all three concentrations of *Nigella sativa* seed extract loaded keratin nanoparticles was found to be 76%, 82% and 64% respectively; likewise, the LC was found to be 55%, 70% and 46 % respectively (Table 2). It was found that 96.81 % EE for thymoquinone loaded poly lactic-co-glycolic acid (PLGA) [32] in earlier studies.

In our results, the concentration of keratin nanoparticles (2mg/mL) and *Nigella sativa* seed extract (1.8mg) was taken for further studies as it exhibited higher E.E and L.C as shown in Table 2. The yield % was higher for KNPs loaded with 1.8 mg NSSE (80%), than other combinations. Like this *Nigella sativa* seed extract, the encapsulated limonella essential oil extract showed 80% yield using solvent evaporation technique [33].

## *In-vitro* pH independent NSSE releases study:

**Figure 3:**
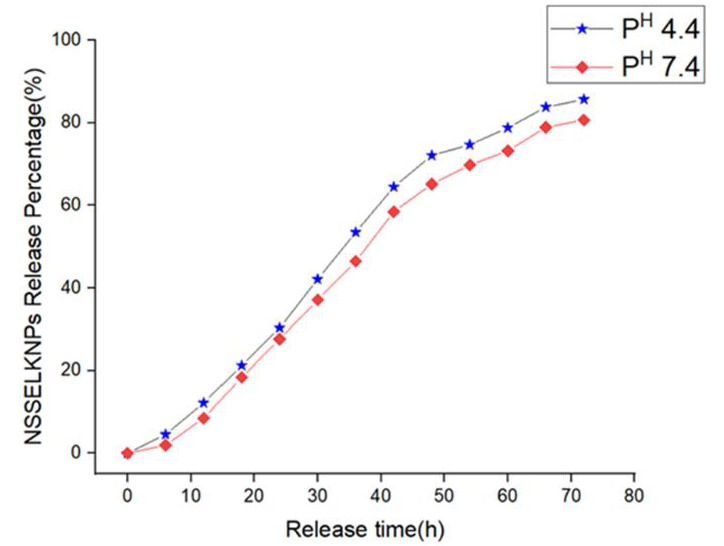
Release Profile of NSSE from the KNPs at different pH.

To analyze the effects of pH responsive behavior and controlled drug delivery, in vitro release profiles of water- based keratin nanoparticles loaded with *Nigella sativa* seed extract (NSSE-KNPs) was investigated under physiological pH 7.4 and pH 4.4. Figure 3 showed in vitro release profiles of NSSE from KNPs over a period of 72 hours. In our results, the release profile pattern occurs from NSSE-KNPs, indicated that there was strong intertwist between NSSE and keratin at pH of 7.4 and 4.4. Nearly 60% of the *Nigella sativa* extract was released within 45 hours and 24% of the remaining extract was released during the next 27 hours. As seen from the release curve, there was less initial burst release and constant, controlled release of NSSE from KNPs take place in acidic media but in basic media there were no initial burst release occurs [34]. This shows there was strong affinity between protein and extract. In earlier report, the controlled release of DOX by keratin nanoparticles was reported in both acidic and basic media [35]. In this pH dependent release study, there was a strong electrostatic interaction between NSSE and the protein which make it to release slowly at pH 7.4 and 4.4 respectively. In our results, the cumulative release of extract during the 72 hours was approximately 84% for NSSE-KNPs at pH 7.4 and 80% at pH 4.4. This result confirmed an initially steady release profile of the keratin nanoparticles followed by steady slow-release rate of *Nigella sativa* seed extract (Figure 3). A maximum of 84 % release of *Nigella sativa* seed extract was recorded after 72 h and this rate increased considerably with time, so there was no much difference in the release rate of the extract at different pH.

**Figure 4:**
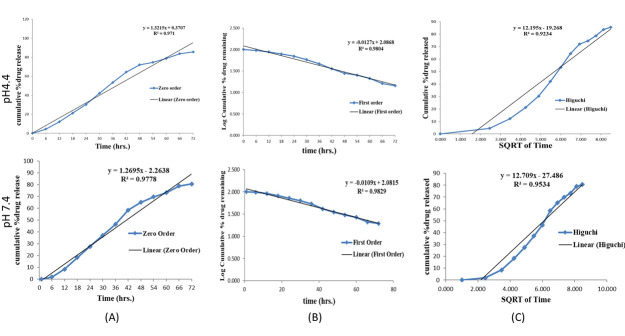
NSSE-KNPs release kinetics curve at pH 4.4 and7.4for A) Zero order, B) First order and C) Higuchi fitting model.

The release empirical kinetic modelsof the *Nigella sativa* seed extract from KNPs were shown in Table 3. The release kinetics of the NSSE from KNPs at acidic and basic pH fits with first order kinetic model (Figure 4 & Table 3) and its R2 value used to evaluate the best fitting model. The R2 value correlates with first order kinetics, which indicates the amount of extract released from keratin nanoparticles was concentration dependent [36]. This showed that the extracts released at a constant rate within 72 hours in both the pH. This may be due to polymer membrane of keratin which was permeable to both *Nigella sativa* seed extract and water. Moreover, their release did not depend on factors like drug composition in the polymeric matrix or in the physiological state. This was mainly due to polymer disentanglement and the erosion, method of preparation, exposure of drug surface area, and dissolution media of the matrix [37].

## SEM:

**Figure 5:**
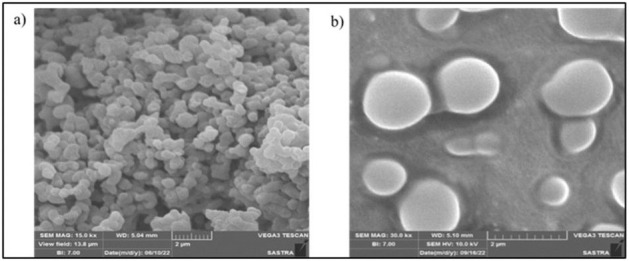
SEM images of (A) Keratin nanoparticles and (B) NSSE extract loaded keratin.

The surface topology of KNPs and NSSE loaded KNPs were shown in Figure 5. The images in the figure showed that synthesized nanoparticles exhibited solid spheres, spherical and smooth shape distribution. This was found in both of KNPs and NSSE loaded KNPs Figures. The conglomerations were observed in KNPs, which was mainly from the interaction between the surface charges of the nanoparticles [38].

## XRD:

**Figure 6:**
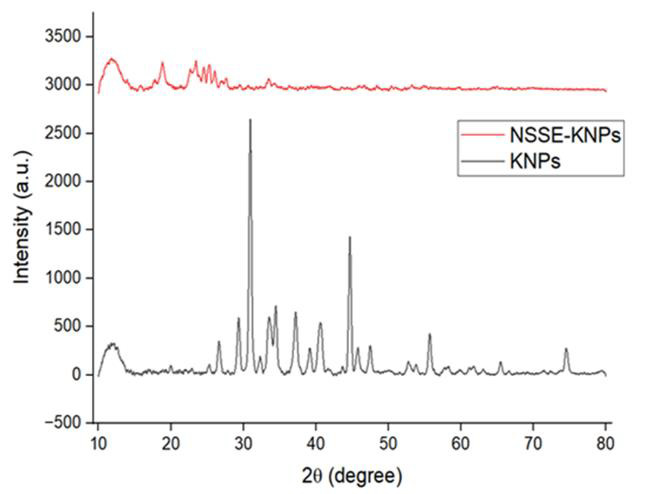
XRD diffract grams of KNPs and NSSE-KNPs.

The XRD patterns of keratin nanoparticles (KNPs) and *Nigella sativa* loaded keratin nanoparticles (NSSE-KNPs)) were shown in the Figure 6. The peaks of KNPs were detected at 2 theta 11.76°, 19.97°, 25.29°, 26.61°, 30.94°, 32.29°, 33.54°, 37.19°, 40.63°, 43.65°, 44.68°, 47.48°, 55.69°, 65.46° and 74.52°. In NSSE-KNPs, the peaks formed at 10.32°, 18.84°, 23.45°, 24.52°, 25.28°, 30.64°, 26.04°, 27.63°, 33.64° and 79.85°. The range between 18° and 22° showed the presence of beta sheet structure in KNPs [39] and NSSE-KNPs [40].

The peak formed around 10 indicated the presence of alpha helix. The main crystalline characteristics of keratin was mainly due to the H bonding formed from inter and intra-molecular interaction. The peaks at 10deg; and 20deg; indicated the presence of alpha and beta helix. The intensity of the peaks confirmed the presence of more beta sheet than alpha helix. The broad peaks at 10deg; and 20deg; showed the partial crystalline character of keratin. The crystalline peaks in keratin were mainly due to the presence of crystalline compounds like proteins, minerals in NSSE. The amorphous peaks showed the presence of non-crystalline components in NSSE like phytochemicals and carbohydrates. Thymoquinone active component present in NSSE also exhibit crystalline peaks.

## FTIR:

**Figure 7:**
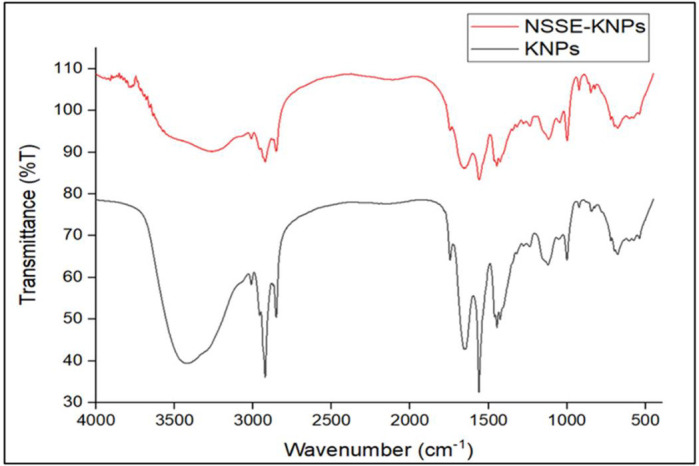
FTIR spectra of KNPs and NSSE-KNPs.

In our results, the characteristic peaks of KNPs and NSSE -KNP appeared at 3402 cm^-1^ and 3266 cm^-1^ which showed the presence of primary amines. The peaks at 1644 cm^-1^ appeared in both KNPs and NSSE-KNPs were identified as (C=O) amide I vibration. This provided information about secondary structure of protein. The peaks at 1545 cm^-1^ and 1561 cm^-1^ in KNPs and NSSE -KNP were corresponds to the N-H bonding. [41]. The bands at 1223cm^-1^ and 1240 cm^-1^ corresponds to amide III vibration. Moreover, the bands of NSSE-KNPs appeared at 1644 cm^-1^ and 1454 cm^-1^ indicated the presence of C=O,C=C and C-H groups respectively. This may be due to predominance of carbon chains in the fatty acids, as *Nigella sativa* oil composed of more than 98% fatty acids [42]. In the NSSE-KNP spectra, few peaks were merged, which indicated the encapsulation of NSSE inside the keratin nanoparticles (Figure 7).

## Anti-microbial activity:

**Figure 8:**
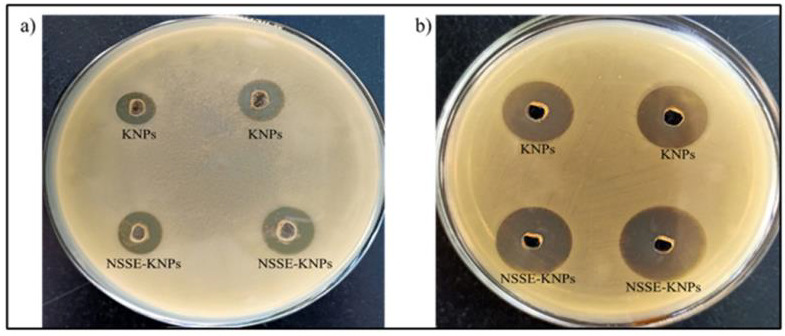
Antibacterial properties shown by KNPs and NSSE-KNPs against a) *Staphylococcus aureus* and b) *Escherichia coli* by well diffusion method

The antimicrobial activity of the nanoparticles was tested on two pathogens which were spread on different Mueller-Hinton agar (MHA) plates. Using sterile cork borer, wells of diameter 6 mm were made in the agar plate. To this, required concentration of drug was loaded. The plates with loaded drug were incubated for 24 hrs at room temperature. The antibacterial activity of keratin nanoparticles and *Nigella sativa* loaded keratin nanoparticles were investigated against common-known pathogenic bacteria such as *Staphylococcus aureus*, *Escherichia coli*. The observed zones of inhibition from different compounds (KNPs and NSSE-KNPs) were measured. The average diameters of inhibition zones with varying concentrations of 5 µl and 10µl for KNPs on *S. aureus* and *E. coli* were 6 mm, 8mm and 12mm, 14mm respectively. For NSSE-KNPs, the inhibition zones were 7mm, 12 mm on *S. aureus* and 14mm, 18 mm on *E. coli* (Figure 8 & Table 4). The NSSE-KNPs showed higher zone of inhibitions for *S. aureus* and *E. coli*. As a result, the antibacterial efficiencies were increased for NSSE-KNPs when compared with KNPs. This is due to the *N. sativa* seed constituents which, was mainly attributed to its phenolic constituents, followed by thymoquinone and its related compounds such as thymohydroquinone, dithymoquinone, and thymol along with carvacrol that plays major role in antimicrobial activity [43, 44].

## Conclusion:

Polymers are quite advantageous in the field of smart drug delivery system which leads to enhanced drug delivery with better pharmacokinetics and therapeutic uses. Biopolymers like protein, polysaccharides, synthetic, semi-synthetic materials, and various natural biomaterials have been prepared in different kinds of nano-formulations for drug delivery applications. In this work, Keratin nanoparticles (KNPs) were first prepared by sonication method followed by encapsulation with Nigella extract(NSSE).The prepared KNPs nanoparticles showed controllable hydrodynamic diameters, fixed poly-dispersity index, negative *zeta potential*, high percentage yield and with encapsulation efficiency above 80%. Moreover, the NSSE-loaded keratin nanoparticles showed high loading efficiency and also exhibited an efficient pH-responsiveness. In both acidic and basic conditions (pH 4.4 & 7.4), the extract was constantly released from the KNPs up to 72 hours. This release data fit very well with the First order of empirical kinetic model. Moreover, other characterization studies like SEM, XRD and FT-IR analysis supported its properties. Thus, water-based keratin nanoparticles demonstrated its potential as drug delivery carrier with plant derived materials and its efficacy to be tested *in vitro* and *in vivo* to prove its valediction in treating various diseases.

## Ethical approval:

The conducted research is not related to either human or animal use.

## Declaration of competing interest:

The authors declare that they have no known competing financial interests or personal relationships that could have appeared to influence the work reported in this paper.

## Figures and Tables

**Table 1:** *Particle size*, PDI values and *zeta potential* measurements of KNPs and NSSE-KNPs at different concentration

**Keratin nanoparticles (mg/mL) in D.H_2_O**	**Amount of *Nigella sativa* Seed extracts (mg)**	***Particle size* (nm)**	**PDI**	**Zeta potential (ZP) (mV)**
1	-	453.6	0.59	-51.2
2	-	338.2	0.41	-43.9
4	-	487.3	0.61	-59.4
1	900	562.7	0.61	-49.9
2	1.8	437.5	0.46	-53
4	3.6	618.4	0.65	-64.4

**Table 2:** Encapsulation efficiency (EE), loading capacity (LC) and yield (%) of *Nigella sativa* seed extract loaded keratin nanoparticles.

**Keratin Nano particles (mg/mL)**	**Amount of *Nigella sativa* Seed extract (mg)**	**Encapsulation efficiency, EE (%)**	**Loading capacity, LC (%)**	**Yield (%)**
1	900	76	55	68
2	1.8	82	70	80
4	3.6	64	46	55

**Table 3:** Parameters and correlation coefficients of empirical kinetic models

**Sample**	**Release condition**	**Zero-order**			**First-order**		**Higuchi**		
		**Q_t_ = K_0_t + C_0_**			**Q_t_ = Q_0_exp(-K_f_^t^)**		**Q_t_ =K_H_^2^√t + C_H_**		
		**K_0_**	**C_0_**	**R_2_**	**K_f_**	**R_2_**	**K_H_**	**C_H_**	**R_2_**
NSSE-KNPs	pH 4.4	1.3215	0.3707	0.971	0.0127	0.9804	12.195	19.268	0.9234
NSSE-KNPs	pH 7.4	1.2695	2.2638	0.978	0.0109	0.9829	12.709	27.486	0.9534

**Table 4:** Antibacterial activity of keratin nanoparticles (KNPs) and *Nigella sativa* loaded keratin nanoparticles (NSSE-KNPs) against *Staphylococcus aureus* and *Escherichia coli*.

**Samples**	***Staphylococcus aureus***		***Escherichia coli***	
	**Zone of inhibition**		**Zone of inhibition**	
Concentration	5µl (mm)	10µl (mm)	5µl (mm)	10µl (mm)
KNPs	6	8	12	14
NSSE-KNPs	7	12	14	18
